# Generation of Bose-Einstein Condensates’ Ground State Through Machine Learning

**DOI:** 10.1038/s41598-018-34725-9

**Published:** 2018-11-05

**Authors:** Xiao Liang, Huan Zhang, Sheng Liu, Yan Li, Yong-Sheng Zhang

**Affiliations:** 1 Laboratory of Quantum Information, University of Science and Technology of China, Hefei, 230026 China; 2 CAS Center for Excellence in Quantum Information and Quantum Physics, University of Science and Technology of China, Hefei, 230026 China; 3 Department of Physics, East China Normal University, Shanghai, 200241 China

## Abstract

We show that both single-component and two-component Bose-Einstein condensates’ (BECs) ground states can be simulated by a deep convolutional neural network. We trained the neural network via inputting the parameters in the dimensionless Gross-Pitaevskii equation (GPE) and outputting the ground-state wave function. After the training, the neural network generates ground-state wave functions with high precision. We benchmark the neural network for either inputting different coupling strength in the GPE or inputting an arbitrary potential under the infinite double walls trapping potential, and it is found that the ground state wave function generated by the neural network gives the relative chemical potential error magnitude below 10^−3^. Furthermore, the neural network trained with random potentials shows prediction ability on other types of potentials. Therefore, the BEC ground states, which are continuous wave functions, can be represented by deep convolutional neural networks.

## Introduction

Because it is difficult to find analytical solutions of non-linear Hamiltonians, investigations of many-body systems rely heavily on numerical simulations. In many-body physics, several methods such as the matrix product state (MPS)^1^ and density matrix renormalization group (DMRG)^2,3^ have shown to be effective in solving for the eigenstates of one-dimensional or two-dimensional chain systems^4^. For systems with more than one dimension, tensor network states^5–10^ and quantum Monte Carlo methods^11–14^ have been widely used.

Currently, artificial intelligence has shown its capability for playing GO^15^. In the last decade, machine learning technology has attracted increasing interest for solving computational problems^16–20^. Several works have investigated accelerating computation with the help of artificial neural networks (ANN), for example, the use of ANN to optimize density-functional theory (DFT) has been intensely investigated^21–25^. Recently, the restricted Boltzmann machine (RBM) has been investigated to find the ground state of spin lattice systems^26^, and the RBM representation ability was further investigated in^27^. Furthermore, the effectiveness of RBM attracts interest for comparing neural network representations to the traditional quantum state representations^28^. In addition to RBM, more advanced neural networks such as convolutional neural network have been shown to effective in distinguishing the phases of many-body systems^29^.

The difference between using the ANN to find a solution of a Hamiltonian and directly solving a Hamiltonian is that ANN accepts inputs and outputs as features and tries to determine the mathematical relationships between these features without using the governing equations. It has been shown that ANN is a powerful approach in pattern recognition problems, such as categorizing a large number of images. The neural network is tested by minimizing the distance between the predicted and real features. The efficiency of the training process depends on both the optimization method and on whether the structure of the neural network is suitable to “learn” the features. It has been shown that the wave functions of lattice systems such as the Ising model and the antiferromagnetic Heisenberg model can be represented by RBM. This naturally raised the question of whether neural networks can represent continuous systems.

Based on quantum mechanics, the wave function contains the complete information regarding a quantum system. Recently, deep convolutional neural networks have been shown to be effective in solving the Schrodinger equation within supervised learning^30^, where the neural network is trained by using the potential field as the input and the ground-state energy as the output. What’s more, machine learning can help to generate Bose-Einstein condensate (BEC) experimentally^31^. We investigate whether the deep convolutional neural network can generate ground-state wave functions. We take the Bose-Einstein condensates^32^ as our example, for which the dynamics are governed by Gross-Pitaevskii equation (GPE)^33–35^. Currently, imaginary time evolution is the main method for numerically solving for the ground state of GPE^36^. Since the initial state is evolving in imaginary time, after many iterations, only the lowest energy part of the initial wave function will dominate. Here, we train the deep convolutional neural networks that generate the ground states of single-component and two-component BECs for both one and two dimensions. Instead of inputting the features and outputting the classification labels, we train the neural network using the GPE parameters as input and the ground-state wave functions as the output.

## Results

### Results for Single-Component BEC

We trained the deep convolutional network with coupling strengths in the range of [0, 500] using 50000 uniformly distributed samples, and in all of the samples, the trapping potential used in the dimensionless GPE is 0.5*x*^2^. The samples were generated using the Trotter-Suzuki code^37^. The ground states are based on the solutions of one-dimensional GPE:

1 $$\mu \psi =[-\frac{1}{2}\frac{{\partial }^{2}}{\partial {x}^{2}}+V(x)+g|\psi {|}^{2}]\psi ,$$

there are 512 points in the position space *x* ∈ [−12, 12], and each sample is obtained after 10^5^ iterations with a time step of 10^−4^. When training the neural network, we randomly select 5000 samples as the validation set, and the remaining 45000 samples are used for training. The distance between the predicted wave function and the original imaginary time evolved wave function is the mean-squared error between the two distributions, and this distance is calculated by $\int |\psi {(x)}_{{\rm{predict}}}-\psi {(x)}_{{\rm{raw}}}{|}^{2}dx$. After training, the distance for either the training set or the validation set is reduced to 10^−5^.

**Figure 1:**
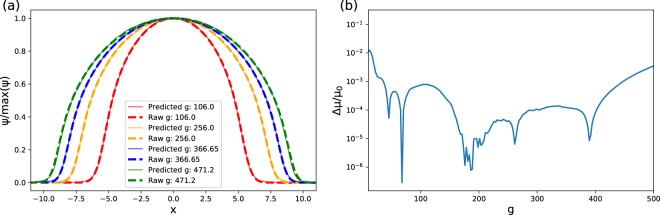
(**a**) Comparisons between the ground-state wave functions generated by the neural network and by imaginary time evolution. Each wave function is normalized relative to its maximum value and we keep 512 points in the *x*-space. (**b**) Comparisons of chemical potentials generated by the neural network and imaginary time evolution. The subfigure depicts the relative chemical potential error |*μ*_predict_ − *μ*_0_|/*μ*_0_.

Our results for the one-dimensional BEC are depicted in Fig. 1. In Fig. 1(a), we compare the wave functions obtained by neural network predictions with imaginary time evolutions. The neural network is trained by the ground states *g* ∈ [0, 500], and it predicts ground states with high precision. To further evaluate the quality of the neural network, we compare the chemical potentials based on the predicted state and on the state obtained by imaginary time evolution. The chemical potentials *μ* were calculated according to: *μ* = 〈*ψ*|*Ĥ*|*ψ*〉/〈*ψ*|*ψ*〉, and are shown in Fig. 1(b). We use the relative chemical potential error |*μ*_predict_ − *μ*_0_|/*μ*_0_ to reveal the quality of the predicted ground states, where *μ*_predict_ is the chemical potential that is calculated for the predicted state and *μ*_0_ is the original chemical potential. As shown in Fig. 1(b), the magnitude of the relative error is on the order of 10^−3^ for most *g* values. In the marginal area of the training set where *g* = 500, the relative *μ* error is 0.0034936 and the *μ* difference is 0.1432696. When *g* = 0, the relative *μ* error is 0.1645099, and the chemical potential is 0.5.

It should be noted that the input into the neural network is only a single parameter g, and the output is the corresponding wave function, so this treatment can be viewed as interpolation. Solving for the ground states in this situation is not sufficient to demonstrate the “learning” ability of neural networks. Therefore, we now benchmark the neural network on one-dimensional arbitrary potentials. We noticed that generating ground-state wave functions of Schrödinger equations has been benchmarked in^38^. In our cases, because of the repulsive interaction between the atoms, the shape of the ground-state wave function is very different from the cases in^38^, and it is crucial to enhance the network’s generation ability under strong interaction and densely distributed arbitrary potentials. In our cases, the trapping potential of the dimensionless GPE is provided by the infinite walls at *x* = ±10, and the arbitrary potential is the Gaussian disorder generated by placing Gaussian functions of width *σ*_*D*_ and random amplitude *A*_*i*_ spaced by equal intervals^39^:

2 $$U(x)=\sum _{i=1}^{{N}_{d}}\,{A}_{i}{e}^{-\frac{{(x-{x}_{i})}^{2}}{2{\sigma }_{D}^{2}}},$$

where *A*_*i*_ is uniformly distributed in the interval [−20, 20], and the distance between adjacent Gaussian functions is 2 × 10^−3^ and *N*_*d*_ = 10^4^.

We trained the neural network on 250000 randomly generated Gaussian disorder potentials under a fixed *σ*_*D*_ and the corresponding ground-state wave functions, with the interaction strength *g* = 1000. The neural network uses the disorder potentials as the inputs and the ground-state wave functions as the outputs, and the mean-squared error between the generated wave functions and raw wave functions is used as the training loss function. Smaller *σ*_*D*_ leads to a more intensely disordered potential and therefore larger variation in the ground-state wave function. Without strong repulsive interactions, the ground state tends to distribute locally even when the amplitude of the disorder potential is strong. Due to the strong repulsive interaction, the ground-state wave function tends to distribute in the entire range of *x*. Therefore, the ground-state wave function is very different from the wave function for the potential without large repulsive interactions. We first trained the neural network on the Gaussian disorder that has *σ*_*D*_ = 0.39. After training, we benchmark the neural network by inputting the potentials that are not in the training dataset.

**Figure 2:**
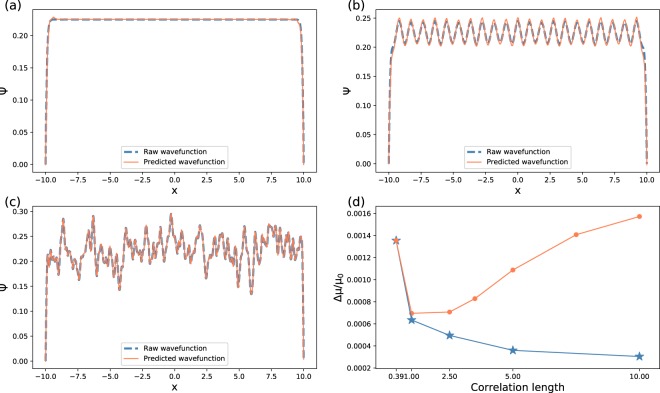
(**a**–**c**) Comparisons between the ground-state wave functions generated by the neural network and by imaginary time evolution under various potentials, where the trapping potential is given by the infinite walls at *x* = ±10. The neural network is trained on the Gaussian disorder that has the correlation length *σ*_*D*_ = 0.39. The ground-state wave functions with (**a**) zero-amplitude disorder input, (**b**) quasiperiodic optical lattice potential, and (**c**) Gaussian disorder with *σ*_*D*_ = 0.39. (**d**) Relative chemical potential (*μ*) errors with respect to the correlation length (*σ*_*D*_) in the Gaussian disorder potentials. The yellow solid circles denote the relative *μ* error calculated from the wave functions generated by the neural network trained on *σ*_*D*_ = 0.39 Gaussian disorders. The blue solid stars denote the relative *μ* error calculated from the wave functions generated by the neural network trained on *σ*_*D*_ = 0.39, 1, 2.5, 5, 10 Gaussian disorders.

The results of the generated ground-state wave functions under several potentials are presented in Fig. 2(a–c). The neural network is trained on the Gaussian disorders with *σ*_*D*_ = 0.39. Figure 2(a) shows the ground-state wave function when the amplitude of the disorder potential is zero, and because of the repulsive interaction, the ground state tends to distribute equally in the entire *x* space, and the relative *μ* error is 1.4 × 10^−3^. Figure 2(b) shows the ground-state wave function when the input potential is a quasiperiodic optical lattice potential, that is formed by combining two incommensurate optical lattices^40^, this potential is useful for generating Anderson localizations^41^. The relative *μ* error is 3.8 × 10^−3^. Figure 2(c) shows the ground-state wave function when the input is a Gaussian disorder within *σ*_*D*_ = 0.39 that is not in the training dataset, and the relative *μ* error is 1.4 × 10^−4^. Figure 2(d) shows the relative chemical potential (*μ*) error with respect to various correlation lengths (*σ*_*D*_) of the Gaussian disorder. As it is depicted by the solid circles, for the neural network trained on Gaussian disorder potentials within *σ*_*D*_ = 0.39, a larger *σ*_*D*_ leads to a less intensive ground-state wave functions, thus, the relative *μ* error in *σ*_*D*_ = 1 is lower than that for *σ*_*D*_ = 0.39. However, when *σ*_*D*_ is too far from the training dataset, the relative *μ* error of the predicted wave functions increases. Meanwhile, the neural network trained on separate *σ*_*D*_ of Gaussian disorder potentials has better accuracy than the neural network trained on *σ*_*D*_ = 0.39 of Gaussian disorder potentials, and the value shown by the solid star at *σ*_*D*_ = 10 is 3 × 10^−4^.

Next, we train the neural network to “learn” two-dimensional states. Here as well, we use the Trotter-Suzuki code to generate the training dataset, in which 50000 samples are prepared in the range of *g* ∈ [0, 500] uniformly, and the position space of interest is the squared area for *x*, *y* ∈ [−7.5, 7.5]. In both *x*- and *y*-direction, 256 points are used, and each sample is obtained after 8000 iterations with the time step of 10^−3^. Since the wave function is two-dimensional, the convolution layers in our neural network are two-dimensional, while the structure of the neural network remains unchanged. The distance to be minimized is then the mean-squared error calculated for the two dimensions. The training process is similar to the one-dimensional conditions. After the training, the mean-squared error for either the training set or the validation set is reduced to the magnitude between 10^−4^ and 10^−5^. We choose the neural network that has the minimum validation error.

**Figure 3:**
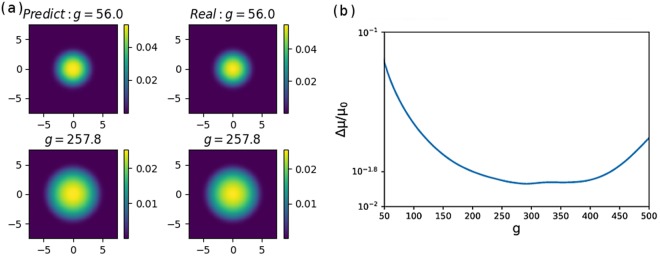
(**a**) Normalized ground-state density distributions generated by the neural network (left column) and by using imaginary time evolution (right column). We keep 256 points for both *x*- and *y*-directions. (**b**) Comparisons of chemical potentials (*μ*) calculated from the wave functions generated by the neural network and by imaginary time evolution. When *g* is close to zero the predicted energy is far from the original energy. The subfigure depicts the *μ* error |*μ*_predict_ − *μ*_0_|/*μ*_0_ where the relative error is in the order of 10^−2^.

Our results for the two-dimensional BECs are shown in Fig. 3. As depicted in Fig. 3(a), the distributions of the neural network predicted states and the states obtained by imaginary time evolution are similar. The relative chemical potential error is depicted in Fig. 3(b). When *g* is close to zero, the spread of the wave function is small compared to our area of interest on the *x* – *y* plane. This makes the training data biased to smaller values, and therefore, the predicted values are smaller than the original value. Estimating the chemical potential using a smaller valued wave function leads to a higher chemical potential, due to the normalization process. Therefore for two-dimensional states, higher coupling strength leads to wider spread of the states, making the predictions of the neural network more accurate.

### Results for Two-Component BEC

We continue to investigate whether a neural network can predict the two-component BEC states. The ground states of two-component BECs are determined by the coupling strengths of each component (*g*_11_ and *g*_22_), the coupling strength between two components (*g*_12_) and the Rabi coupling coefficient (Ω). The dimensionless GPE of a two-component BEC given by

3 $$\mu [\begin{array}{c}{\psi }_{1}\\ {\psi }_{2}\end{array}]=[\begin{array}{cc}{H}_{1} & \frac{{\rm{\Omega }}}{2}\\ \frac{{\rm{\Omega }}}{2} & {H}_{2}\end{array}]\,[\begin{array}{c}{\psi }_{1}\\ {\psi }_{2}\end{array}],$$

where *H*_1_ and *H*_2_ are:

4 $$\begin{array}{c}{H}_{1}={T}_{1}+{V}_{1}+{g}_{11}|{\psi }_{1}{|}^{2}+{g}_{12}|{\psi }_{2}{|}^{2},\\ {H}_{2}={T}_{2}+{V}_{2}+{g}_{22}|{\psi }_{2}{|}^{2}+{g}_{12}|{\psi }_{1}{|}^{2},\end{array}$$

where *T*_1(2)_ is the kinetic energy and *V*_1(2)_ is the potential. To demonstrate the capability of the neural network, we investigate the ground states in the range of Ω ∈ [−20, 0], while *g*_11_, *g*_12_, *g*_22_ = 100(1.03, 1, 0.97). Since there are two components, our neural network must output two distributions for each input of Ω.

**Figure 4:**
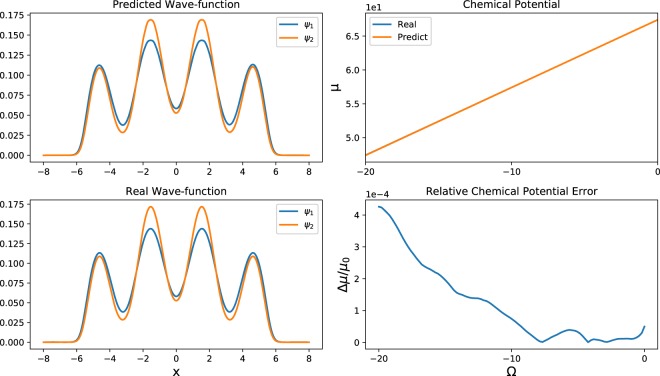
For Ω = −1, the states predicted by neural network and the real states are depicted in the left column. In the *x*-direction we keep 512 points. In the right column, the chemical potentials and the relative chemical potential errors are depicted in the range of Ω ∈ [−20, 0].

First, we train the neural network using one-dimensional states. The potential is *V*(*x*) = 0.5*x*^2^ + 24cos^2^*x* and our area of interest is *x* ∈ [−8, 8] with 512 points. Since the range of Ω ∈ [−20, 0] is small, we prepare 13000 samples using the Trotter-Suzuki code, with each sample generated after 10^5^ iterations with a time step of 10^−4^. Since the wave function changes faster as Ω approaches zero, in addition to sampling 10000 points uniformly in the range of Ω ∈ [−20, 0], we sample 3000 points in the range of Ω ∈ [−2, 0]. Moreover, 1300 samples are randomly picked as the validation set. After training the neural network, the mean-squared error for both the training set and the validation set has the magnitude of 10^−6^. In Fig. 4, it is shown that for Ω = −1, the predicted wave functions are identical to the real wave functions. To quantify the quality of the predicted wave function, we compare the chemical potentials calculated by these wave functions. The chemical potential of the components is calculated as

5 $$\mu =[\begin{array}{cc}{\psi }_{1} & {\psi }_{2}\end{array}][\begin{array}{cc}{H}_{1} & \frac{{\rm{\Omega }}}{2}\\ \frac{{\rm{\Omega }}}{2} & {H}_{2}\end{array}]\,[\begin{array}{c}{\psi }_{1}\\ {\psi }_{2}\end{array}].$$

As depicted in Fig. 4, the relative chemical potential error is on the order of 10^−4^. In the marginal area where Ω is close to −20, the relative error increases due to the lack of samples. Because of the additional 3000 samples, the energy error remains low for Ω close to zero.

**Figure 5:**
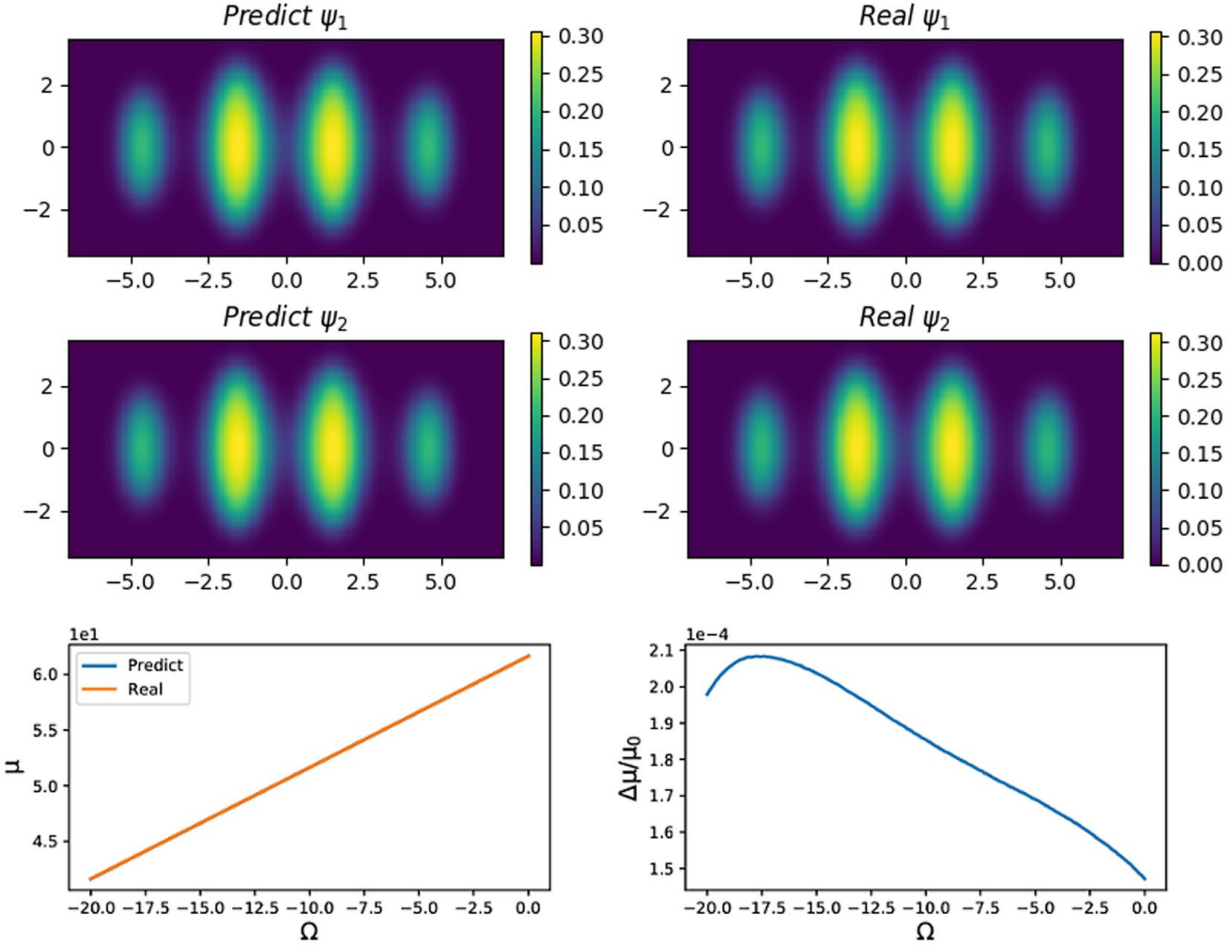
For 2-dimensional BECs, when Ω = −3.12, both *ψ*_1_ and *ψ*_2_ are predicted by the neural network are nearly identical to the real wave functions. We keep 256 points for the *x* direction and 128 points for the *y* direction. The relative chemical potential error is on the order of 10^−4^ for all possible Ω.

Figure 5 depicts the two-dimensional conditions. The neural network is also trained using 13000 samples with 1300 samples used for validation, and each sample is generated by 8000 iterations with a time step of 10^−3^. The potential is *V*(*x*, *y*) = 0.5(*x*^2^ + 5*y*^2^) + cos^2^*x*. Since the confinement in the *y*-direction is stronger than that in the *x*-direction, our area of interest is *x* ∈ [−7, 7] with 256 points and *y* ∈ [−3.5, 3.5] with 128 points. As depicted in the figure, for Ω = −3.12, the predicted states are nearly identical to the real states. The relative chemical potential error in the range of Ω ∈ [−20, 0] is on the order of 10^−4^.

Why are the chemical potential errors lower than that of the single-component BEC? This is because we use 11700 samples in the small range of Ω ∈ [−20, 0], the dataset is more dense than that used for the single-component BEC.

## Methods

The dynamics of a 2-dimensional BEC are governed by the following GPE,

6 $$i\hslash \frac{\partial }{\partial t}\psi =[-\frac{{\hslash }^{2}}{2m}({\partial }_{x}^{2}+{\partial }_{y}^{2})+{V}_{{\rm{trap}}}\,(x,y)+{V}_{{\rm{disorder}}}(x,y)+g|\psi {|}^{2}]\psi ,$$

where we consider the dimensionless equation with *m* = 1 and ℏ = 1, under a dimensionless harmonic potential ${V}_{{\rm{trap}}}(r)=\frac{1}{2}({x}^{2}+{y}^{2})$. We take the normalization $\int |{\rm{\Psi }}{|}^{2}dxdy=1$ in this paper. The training dataset is generated by the imaginary evolution governed by GPE. Since the ground state of GPE is a real function, the weights of the neural network are real numbers. Meanwhile the outputs of the neural network are real distributions.

The dynamics of a two-component BEC is governed by

7 $$i\hslash \frac{\partial }{\partial t}[\begin{array}{c}{\psi }_{1}\\ {\psi }_{2}\end{array}]=[\begin{array}{cc}{H}_{1} & \frac{{\rm{\Omega }}}{2}\\ \frac{{\rm{\Omega }}}{2} & {H}_{2}\end{array}]\,[\begin{array}{c}{\psi }_{1}\\ {\psi }_{2}\end{array}],$$

where *H*_1_ and *H*_2_ are defined in Eq. (4). We take the normalization $\int |{\psi }_{1}{|}^{2}+|{\psi }_{2}{|}^{2}d\tau =1$ where *τ* denotes the total position space. The training dataset is generated by the imaginary evolution governed by GPE. Under each coupling strength of Ω, the neural network outputs two wave-functions, each corresponds to one component.

We set up a deep convolutional neural network to learn the ground states of one-dimensional and two-dimensional GPEs. A convolutional neural network uses filters to scan the feature surface, and the relationships between the adjacent feature sites can be efficiently “learned” by several filters scanning simultaneously. When the neural network contains tens to hundreds of convolution layers, the obtained deep convolutional network excels at pattern recognition tasks such as image classification, speech recognition and language translation.

**Figure 6:**
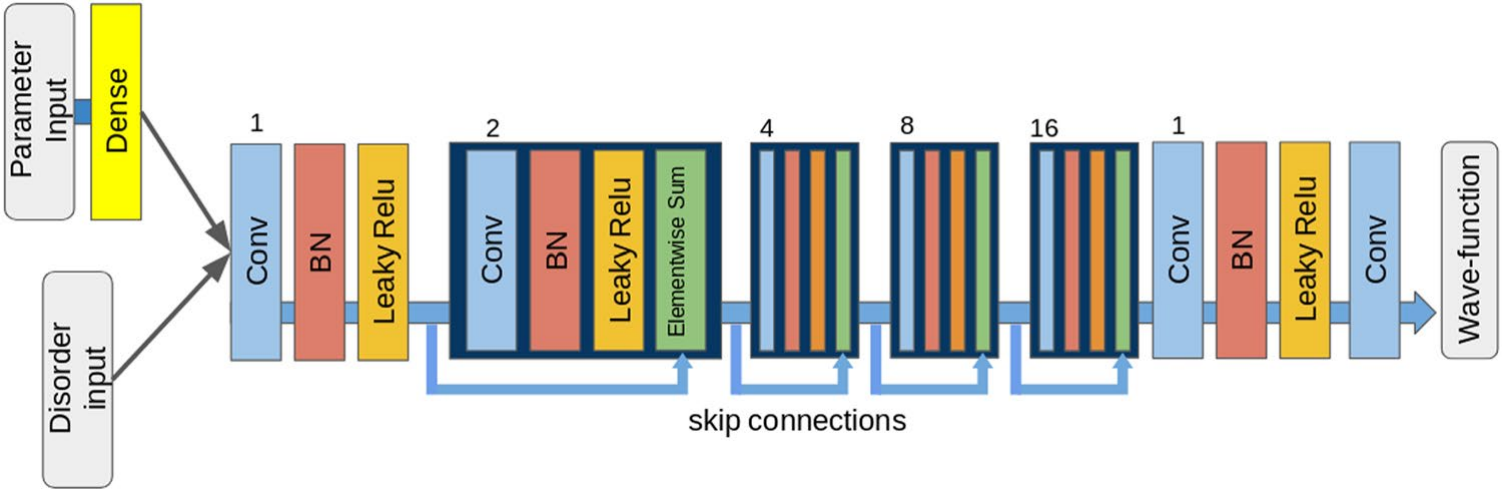
Our neural network consists of stacks of convolutional blocks. Each stack consists of a convolutional layer, batch normalization and the Leaky-ReLU activation. The convolution layers in the first and the last blocks have the dilation rate of unity, and the convolution layers in the intermediate blocks have the dilation rate of 2, 4, 8 and 16. A dilation rate greater than unity is beneficial for learning the wave functions at the larger scale. When the input is the repulsive interaction strength *g* for single component BEC or the coupling strength Ω for two-component BECs, the input is transformed into a high-dimensional vector by a dense layer. When the input is an arbitrary potential, the arbitrary potential is fed directly into the first convolution layer. The last convolution layer outputs the ground-state wave function, and it has the dilation rate of unity. The number of the output channels for the last convolution layer is either one for single-component BEC or two for two-component BECs. Since the network is deep, we use elementwise sum after each block to keep the gradients flowing properly.

The neural network structure used in this paper is presented in Fig. 6. The main part of the neural network consists of seven convolutional layers, while each convolution layer is followed by batch normalization (BN) and Leaky-ReLU non-linear activation. To keep the gradients flowing properly, after each convolution block the total output of the block is the summation of the block input and the block output. The input and output channel in the convolution layer is 64 except the first and the last convolution layer. Comparing with dense connected layers, the last convolution layer is crucial to generate the high quality ground-state wave functions, as the convolution filters are grasping the regional features, larger filters are helpful to generate smoother wave functions. In our cases the convolution filter size is chosen to be 10. The output of the neural network is the ground-state wave function. The input of the neural network varies based on the kinds of problems. In the training the neural network on ground-state wave functions with respect to the repulsive interaction strength *g* or the coupling strength Ω, there is only one input, namely, this one-dimensional input is transformed into a high-dimension vector by a dense layer, as shown in the figure. In the training of the neural network on the ground-state wave functions with respect to arbitrary potentials, the input itself is a high-dimensional vector and thus can be fed directly into a convolutional layer. The formation of the inputs and outputs depends on the problem, and since the neural network is built layer by layer, the structure of the neural network is very flexible. The training of the neural network is performed efficiently using modern graphics processing units.

## Conclusion

We have shown that continuous wave functions like the ground-states of BEC can be “learned” and simulated by deep convolution neural networks. Besides the fact that latticed systems can be simulated by neural networks like RBM, since that convolution network is good at grasping relations between adjacent features, here we show the systems with continuous and smooth distributions can be simulated by convolution neural networks.

The convolution neural network we trained predicts ground states in high precisions when the inputting coupling strength is in the range of the training set. When inputting a value which is not in the training set such as *g* = 550, the relative error of the predicted energy is still in the magnitude of 10^−3^ (The generated wave function and the intermediate outputs are depicted in Figure S1 in the supplementary information). Although the effectiveness of our neural network depends on the training set, the neural network can be a fast BEC ground states generator. After training, the neural network predicts ground states much faster than imaginary time evolutions. Especially for two-dimensional cases, predicting a two-component BEC using neural network takes less than a millisecond while the imaginary time evolution for 8000 iterations takes about 6 seconds on the same Graphics Processing Unit (GPU).

Furthermore, we have benchmarked the same neural network on inputting Gaussian disorder potentials. The neural network trained on the Gaussian disorder within *σ*_*D*_ = 0.39 can predict the ground-state wave functions on other kinds of potentials. Therefore, the neural network is not simply “remembering” the mapping between the input potential and the output wave function. By training, the neural network finds a new method to solve GP equations (The intermediate outputs of the neural network is depicted in Figure S1 in the supplementary information).

The effectiveness of convolutional neural network for describing continuous quantum system raises some open questions. Since the ground states can be “learned” and generated by deep convolutional neural networks, can we solve GPE without training, just having the knowledge that the ground state can be represented by the neural networks?

## Electronic supplementary material

Supplementary Information

## References

[1] Garcia DP, Verstraete F, Wolf MM, Cirac JI (2007). Matrix product state representations. Quantum Inf. Comput..

[2] Schollwoeck U (2005). The density-matrix renormalization group. Rev. Mod. Phys..

[3] Schollwoeck U (2011). The density-matrix renormalization group in the age of matrix product states. Annals of Physics.

[4] Stoudenmire EM, White SR (2012). Studying Two Dimensional Systems With the Density Matrix Renormalization Group. Annu. Rev. Conden. Ma. P..

[5] Orus R (2014). A Practical Introduction to Tensor Networks: Matrix Product States and Projected Entangled Pair States. Annals of Physics.

[6] Evenbly G, Vidal G (2011). Tensor network states and geometry. J. Stat. Phys..

[7] Singh S, Pfeifer RNC, Vidal G (2011). Tensor network states and algorithms in the presence of a global U (1) symmetry. Phys. Rev. B.

[8] Singh S, Vidal G (2012). Tensor network states and algorithms in the presence of a global SU (2) symmetry. Phys. Rev. B.

[9] Xie ZY, Jiang HC, Chen QN, Weng ZY, Xiang T (2009). Second renormalization of tensor-network states. Phys. Rev. Lett..

[10] Zhao HH (2010). Renormalization of tensor-network states. Phys. Rev. B.

[11] Jarell M (1992). Hubbard model in infinite dimensions: A quantum Monte Carlo study. Phys. Rev. Lett..

[12] Astrakharchik, G. E., Boronat, J., Casulleras, J. & Giorgini, S. Equation of state of a Fermi gas in the BEC-BCS crossover: A quantum Monte Carlo study *Phys. Rev. Lett*. **93** (2004). 10.1103/PhysRevLett.93.200404 15600904

[13] Hohenadler M, Lang TC, Assaad FF (2011). Correlation effects in quantum spin-hall insulators: A quantum monte carlo study. Phys. Rev. Lett..

[14] Makivic MS, Ding HQ (1991). Two-dimensional spin-1/2 Heisenberg antiferromagnet: A quantum Monte Carlo study. Phys. Rev. B..

[15] Silver D (2017). Mastering the game of Go without human knowledge. Nature.

[16] Yao K, Parkhill J (2016). Kinetic energy of hydrocarbons as a function of electron density and convolutional neural networks. J. Chem. Theory. Comput..

[17] Caetano C, Amorim JL, Lemes MR, Pino AD (2011). Using neural networks to solve nonlinear differential equations in atomic and molecular physics. Int. J. Quantum. Chem..

[18] Li, H. Z. *et. al*. *An* Accurate and Efficient Method to Predict Y-NO Bond Homolysis Bond Dissociation Energies. *Math. Probl. Eng*. (2013).

[19] Montavon G (2013). Machine learning of molecular electronic properties in chemical compound space. New. J. Phys..

[20] Monterola C, Saloma C (2001). Solving the nonlinear Schrodinger equation with an unsupervised neural network. Opt. Express..

[21] Snyder JC, Rupp M, Hansen K, Muller KR, Burke K (2012). Finding Density Functionals with Machine Learning. Phys. Rev. Lett..

[22] Brockherde F, Li L, Burke K, Muller KR (2017). By-passing the Kohn-Sham equations with machine learning. Nat. Commun..

[23] Rupp M, Tkatchenko A, Muller KR, Lilienfeld OA (2012). Fast and Accurate Modeling of Molecular Atomization Energies with Machine Learning. Phys. Rev. Lett..

[24] Seko A, Maekawa T, Tsuda K, Tanaka I (2014). Machine learning with systematic density-functional theory calculations: Application to melting temperatures of single- and binary-component solids. Phys. Rev. B.

[25] Behler J, Parrinello M (2007). Generalized Neural-Network Representation of High-Dimensional Potential-Energy Surfaces. Phys. Rev. Lett..

[26] Carlo G, Troyer M (2017). Solving the quantum many-body problem with artificial neural networks. Science.

[27] Deng DL, Li XP, Sarma SD (2017). Quantum entanglement in neural network states. Phys. Rev. X.

[28] Glasser, I., Pancotti, N., August, M., Rodriguez, I. D. & Cirac, J. I. Neural Networks Quantum States String-Bond States and chiral topological states. *arXiv:1710.04045v1* (2018).

[29] Ch’ng K, Carrasquilla J, Melko RG, Khatami E (2017). Machine Learning Phases of Strongly Correlated Fermions. Phys. Rev. X.

[30] Mills K, Spanner M, Tamblyn I (2017). Deep learning and the Schrödinger equation. Phys. Rev. A..

[31] Wigley PB (2016). Fast machine-learning online optimization of ultra-cold-atom experiments. Sci. Rep..

[32] Durfee DS, Ketterle W (1998). Experimental studies of Bose-Einstein condensation. Opt. Express.

[33] Bao W, Jaksch D, Markowich PA (2003). Numerical solution of the Gross-Pitaevskii equation for Bose-Einstein condensation. J. Comput. Phys..

[34] Ananikian D, Bergeman T (2006). Gross-Pitaevskii equation for Bose particles in a double-well potential: Two-mode models and beyond. Phys. Rev. A.

[35] Lieb, E. H., Seiringer, R. & Yngvason, J. Bosons in a trap: A rigorous derivation of the Gross-Pitaevskii energy functional. *The Stability of Matter: From Atoms to Stars* 685–697 (Springer Berlin Heidelberg, 2001).

[36] Chiofalo ML, Succi S, Tosi MP (2000). Ground state of trapped interacting Bose-Einstein condensates by an explicit imaginary-time algorithm. Phys. Rev. E.

[37] Massively Parallel Trotter-Suzuki Solver, https://trotter-suzuki-mpi.github.io.

[38] Steinke, S. Solving the Schrödinger equation with deep learning, https://becominghuman.ai/solving-schr%C3%B6dingers-equation-with-deep-learning-f9f6950a7c0e (2017).

[39] Donsa S, Holfstätter H, Koch O, Burgdörfer J, Brêzinová I (2017). Long-time expansion of a Bose-Einstein condensate: Observability of Anderson localization. Phys. Rev. A.

[40] Roati G (2013). Anderson localization of a non-interacting Bose-Einstein condensate. Nature.

[41] Zhou L, Pu H, Zhang W (2013). Anderson localization of cold atomic gases with effective spin-orbit interaction in a quasiperiodic optical lattice. Phys. Rev. A.

