# Supplementary material for: Generation of Bose-Einstein Condensates’ Ground State Through Machine Learning
Source: Sci Rep. 2018 Nov 5;8:16337. doi: 10.1038/s41598-018-34725-9 (PMC6218512; doi:10.1038/s41598-018-34725-9)
Supplement: Supplementary file 1 — Supplementary Information [file 41598_2018_34725_MOESM1_ESM.pdf]

Supplementary Information for:

Generation of Bose-Einstein Condensates' Ground State Through Machine Learning

Xiao Liang[1,2], Huan Zhang[1,2], Sheng Liu[1,2], Yan Li[3,*] and Yong-Sheng Zhang[1,2,+]

1. Laboratory of Quantum Information, University of Science and Technology of China, Hefei, 230026, China
2. Synergetic Innovation Center of Quantum Information and Quantum Physics, University of Science and Technology of China, Hefei, 230026, China
3. Department of Physics, East China Normal University, Shanghai, 200241, China

*E-mail: yli@phy.ecnu.edu.cn
+E-mail:yshzhang@ustc.edu.cn

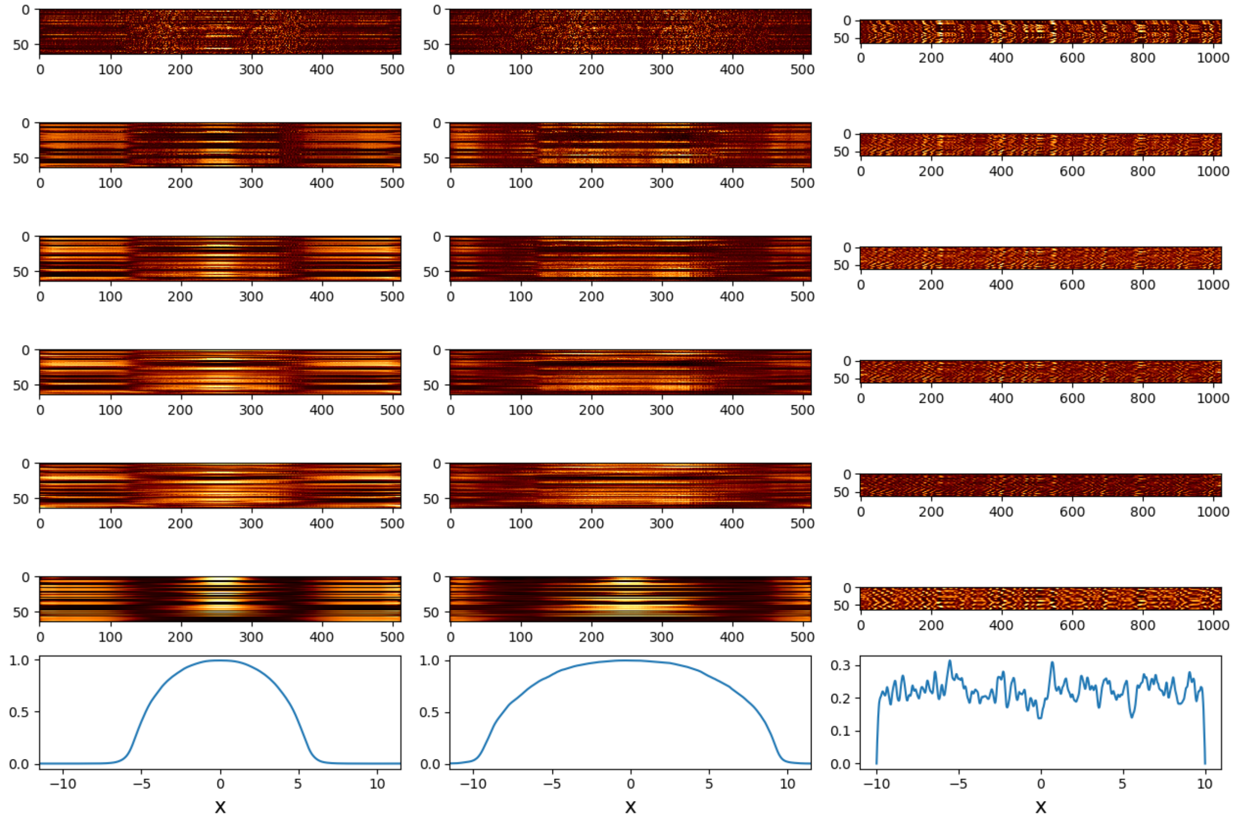

Figure S1: The intermediate outputs after each convolution layer in the deep convolutional neural network. The left column and the middle column depict the generation processes when the inputs g=50 and g=550 respectively. The right column depicts the generation process when the input is the Gaussian disorder within σ_D=0.39. As revealed by the left column and the middle column, the neural network firstly identifies the width of the ground-state wave function, then polish the outputs layer by layer. Because of the skip connections, each output from the second to the fourth convolution layer is mixed with the previous layer's output, which is clearly revealed by the figure. Although g=550 is not in the training dataset, the intermediate outputs has the same patterns as that when g=50. The relative chemical potential error for g=550 is $7\text{x}10^{-3}$.
